# Kink of Subclavian Artery Mimicking Stenosis

**DOI:** 10.1155/2016/4274936

**Published:** 2016-12-12

**Authors:** Hatice S. Kemal, Aziz Gunsel, Murat Kocaoglu, Levent Cerit, Hamza Duygu

**Affiliations:** ^1^Department of Cardiology, Near East University Hospital, Nicosia, Cyprus; ^2^Department of Radiology, Near East University Hospital, Nicosia, Cyprus

## Abstract

The treatment for subclavian artery stenosis includes the more common endovascular therapy rather than surgical intervention in symptomatic patients. We present a case of a 79-year-old man with coronary artery bypass graft where subclavian artery stenosis was found incidentally. In this asymptomatic case, we have merged clinical and multiple imaging modalities to secure the diagnosis and treatment plan.

## 1. Introduction

Subclavian artery stenosis (SAS), caused by atherosclerotic occlusive plaques, is most often asymptomatic and does not require specific therapy, other than that directed at the underlying etiology [1]. The most important point on deciding the type of treatment is whether the patient has ischemic symptoms or is asymptomatic. Symptomatic cases are treated primarily with interventional modalities. Herein, we report a case of asymptomatic SAS discovered incidentally in a patient with coronary-artery bypass graft (CABG).

## 2. Case Report

**Figure 1:**
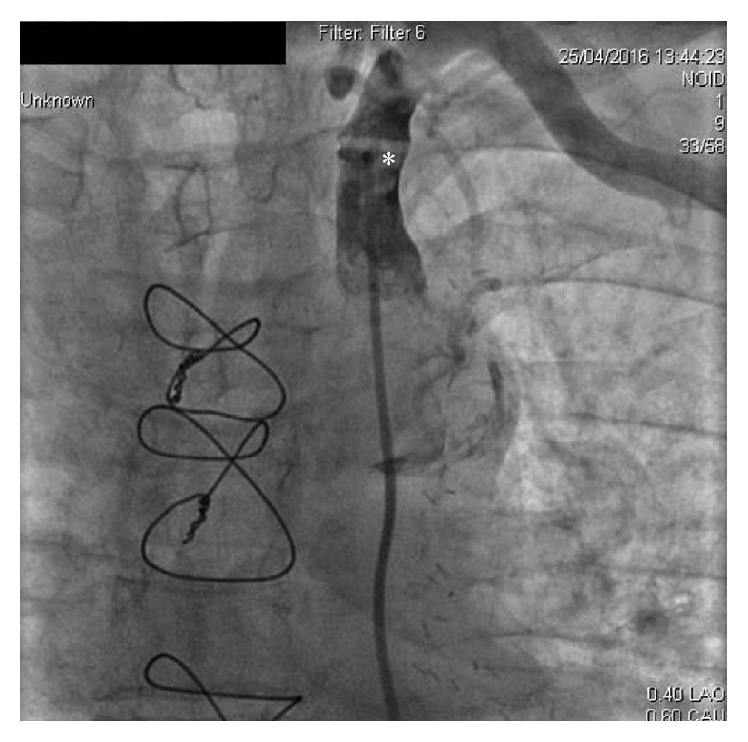
A web-like calcification (asterisk) at the proximal segment of the left subclavian artery seen at angiography.

**Figure 2:**
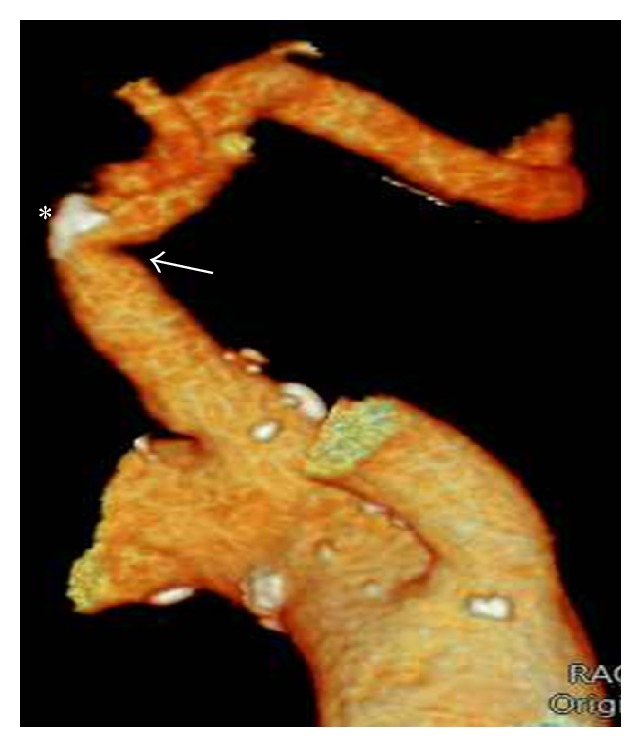
Computed tomography angiography revealing calcified plaque (asterisk) and a kink (arrow) just before the atherosclerotic plaque.

A 79-year-old man who had undergone CABG 10 years earlier and had a history of hypertension and dyslipidemia was admitted to the outpatient clinic with stable angina pectoris. Coronary angiography was performed via right femoral access and aortography was performed to localize venous bypass grafts. Angiography of the left internal mammary artery (LIMA) was done through the left subclavian artery using JR4 (Medtronic Pro-Flo® 6F) diagnostic coronary catheter. At first, the left subclavian artery could not be traversed with the catheter, soft coronary J guidewire (0.014 inches), forming a loop at the proximal segment as if the artery was totally occluded. Administration of contrast agent revealed a web-like calcified lesion at the proximal segment of the artery, creating a firm line between the distal segments (Figure 1). The J wire was then exchanged for an exchange-length hydrophilic wire (0.014 inches) and the web-like calcified segment was passed allowing selective LIMA cannulation and TIMI 3 flow was detected with no flow reversal suggestive of coronary steel. Angiography was completed with full cannulation of all native and bypass graft arteries. The patient was then examined for SAS and had no symptoms of intermittent arm claudication, cold left upper limb, or systolic blood pressure difference between two arms. Computed tomography angiography confirmed a calcified plaque causing less than 20% stenosis at the proximal segment of the left subclavian artery and a kink formation just before the atherosclerotic plaque (Figure 2). It was recommended that the patient undergo aggressive atherosclerotic risk factors modification and regular follow-up.

## 3. Discussion

SAS is usually asymptomatic and is essentially related to atherosclerosis. Other etiologies such as traumatic or congenital causes and inflammatory diseases are rare [2]. It is recommended that symptomatic SAS be treated with endovascular stenting and angioplasty as first-line management. In cases where intervention is not suitable or is unsuccessful, open surgery is suggested [3]. The surgical treatment is either endarterectomy or carotid subclavian bypass, which both carry a low morbidity and mortality risk, although the risk is higher than in an endovascular approach.

In patients with a history of CABG, coronary subclavian steal was reported as flow reversal in the internal mammary artery graft [4] and recurrent ischemia and even myocardial infarction have been reported [5]. Salman et al. have described a case series of SAS and different modalities of intervention [6]. Swaminathan et al. have described a case of SAS causing low flow state in brachiocephalic AVF that was treated with percutaneous intervention [7]. The most important point is whether the patient has symptoms: SAS due to flow reversal of the vertebral artery, ischemic symptoms such as pallor, pain, paraesthesia, and coldness of the arm, left upper limb claudication, pulse deficit, or neurological symptoms.

Clinical examination and blood pressure difference between the two arms are important. Although the use of computed tomography angiogram is rapidly increasing, angiography is the gold standard for diagnosis. Saleem and Majdi have described a case where left SAS was diagnosed with echocardiography by recording a continuous flow with fast systolic and slow diastolic components at aortic arch-descending aorta junction [8].

For the time being, not much is known about the progression of SAS, and investigation and invasive treatment are only recommended in symptomatic patients, whereas aggressive risk factor modification is recommended in asymptomatic patients [3], since it is known that SAS is highly associated with subclinical atherosclerosis [9]. This case highlights the importance of thorough investigation and combining information from multiple imaging modalities to secure the diagnosis and treatment plan.
